# Fatal concentrations of antidepressant and antipsychotic drugs in postmortem femoral blood

**DOI:** 10.1093/jat/bkad044

**Published:** 2023-07-13

**Authors:** Pirkko Kriikku, Ilkka Ojanperä

**Affiliations:** Forensic Toxicology Unit, Finnish Institute for Health and Welfare (THL), P.O. Box 30, Mannerheimintie 166, Helsinki FI-00271, Finland; Department of Forensic Medicine, University of Helsinki, P.O. Box 21, Haartmaninkatu 3, Helsinki FI-00014, Finland; Forensic Toxicology Unit, Finnish Institute for Health and Welfare (THL), P.O. Box 30, Mannerheimintie 166, Helsinki FI-00271, Finland; Department of Forensic Medicine, University of Helsinki, P.O. Box 21, Haartmaninkatu 3, Helsinki FI-00014, Finland

## Abstract

Antidepressants and antipsychotics are both an important class of prescription drugs within postmortem (PM) toxicology because most of the substances are toxic in overdose and the mental disorders being treated may be associated with suicidality. A wide range of antidepressants and antipsychotics are currently included in up-to-date PM toxicology analysis protocols. However, apart from case studies, few reports on fatal concentrations based on large number of cases have been published in the literature. Based on PM investigations in Finland between 2000 and 2020, this study provides fatal reference concentrations in poisonings due to an antidepressant or an antipsychotic drug assigned as the principal intoxicant. Summary statistics for drug concentrations in PM femoral blood (min, max, mean, 10th, 25th, 50th, 75th, 90th percentile) were calculated for 17 antidepressant (*N* = 2,007) and for 12 antipsychotic drugs (*N* = 1,161). The proportion of suicide, accident and undetermined manner of death is indicated for each drug. Further, the fatal concentrations obtained in this study were evaluated by comparison with fatal and “normal” PM concentrations reported by two previously published approaches, the grouped causes of death approach and the all causes of death approach, respectively. This study shows that, despite the well-known variation in PM drug concentrations, competently generated fatal concentration results for the drugs studied are consistent to such an extent that they can be used as a reference in the interpretation process.

## Introduction

Approximately 30 antidepressant drugs and the same number of antipsychotic drugs are currently used as prescription medicines mainly for treating mental illnesses but also for a variety of somatic diseases and symptoms. In addition to the accepted indications, many drugs are used off-label for a variety of indications for which they are not officially approved (1, 2). Antidepressants and antipsychotics entered the market in the 1950s, and some of these early substances are still in medical use. Along with opioids and illicit drugs, antidepressant and antipsychotic drugs are important groups of substances in postmortem (PM) toxicology, because they are associated with an above-average number of poisoning deaths (3). The mental disorders being treated and occasionally the medication used may be associated with suicidality.

The types of antidepressants include tricyclic antidepressants (TCA), such as amitriptyline, clomipramine, doxepin, nortriptyline and trimipramine, selective serotonin reuptake inhibitors (SSRI), such as citalopram, fluoxetine, paroxetine and sertraline, serotonin and norepinephrine reuptake inhibitors, such as duloxetine and venlafaxine, monoamine oxidase inhibitors, such as moclobemide, and atypical antidepressants, such as bupropion, mianserin, mirtazapine and trazodone, which affect one or several neurotransmitters. The main clinical indication for antidepressant treatment is depression, followed by stress, anxiety, pain and sleep disorders (4).

Antipsychotics can be divided into first-generation antipsychotics (typical antipsychotics), such as chlorpromazine, chlorprothixene and levomepromazine, and second-generation antipsychotics (atypical antipsychotics), such as clozapine, olanzapine and quetiapine. Pharmacologically, the first-generation antipsychotics are mainly dopamine receptor antagonists while the second-generation antipsychotics are mainly serotonin-dopamine antagonists. In addition to psychotic illnesses, antipsychotics are used for treating acute mania, agitation, treatment-resistant schizophrenia, schizoaffective disorder, bipolar disorder, Tourette syndrome and hyperactivity (5).

Reference concentrations of drugs play a crucial role in the interpretation of PM toxicology results. However, due to the PM drug redistribution phenomenon (6–8), tables of standard clinical plasma concentration data (9) are of limited value as PM drug concentrations are not necessarily the same as the antemortem (AM) concentrations prior to death. Although PM reference values in cases of fatal poisoning have been published, the data in case notes and compilations of cases are heterogeneous in terms of blood sampling site, the lower limit of quantification (LLOQ) and the number of cases involved (10, 11).

**Table I.** Drug Analysis Methods with the Range of Lower Limits of Quantification Applied to Antidepressant and Antipsychotic Drugs in Postmortem Femoral Blood. For Comparison, Therapeutic Reference Concentrations in Living Subjects’ Plasma are Given

Substance		Method^a^	LLOQ (mg/L)^b^	Therapeutic concentration in plasma (mg/L)^c^
Antidepressants	Amitriptyline	GC–NPD, UPLC–PDA-CAD	0.1	0.3
	Bupropion	GC–NPD, LC–QqQ	0.001–0.1	0.02
	Citalopram	GC–NPD, UPLC–PDA-CAD	0.1	0.11
	Clomipramine	GC–NPD, UPLC–PDA-CAD	0.1	0.25 (–0.4)
	Doxepin	GC–NPD, UPLC–PDA-CAD	0.05	0.2
	Duloxetine	LC–QqQ, UPLC–PDA-CAD	0.01–0.05	0.12
	Fluoxetine	GC–NPD, UPLC–PDA-CAD	0.1–0.2	0.5
	Fluvoxamine	LC–QqQ, UPLC–PDA-CAD	0.03–0.1	0.23
	Mianserin	GC–NPD, UPLC–PDA-CAD	0.05–0.1	0.07
	Mirtazapine	GC–NPD, UPLC–PDA-CAD	0.05–0.1	0.08 (–0.3)
	Moclobemide	GC–NPD, UPLC–PDA-CAD	0.05–0.1	1.0 (–3)
	Nortriptyline	GC–NPD, UPLC–PDA-CAD	0.1	0.17
	Paroxetine	LC–QqQ, UPLC–PDA-CAD	0.004–0.1	0.065
	Sertraline	GC–NPD, UPLC–PDA-CAD	0.05–0.1	0.15 (–0.5)
	Trazodone	GC–NPD, LC–QqQ	0.01–0.2	1.0 (–2)
	Trimipramine	GC–NPD, UPLC–PDA-CAD	0.1	0.3
	Venlafaxine	GC–NPD, UPLC–PDA-CAD	0.1	0.4
Antipsychotics	Amisulpiride	UPLC–PDA-CAD	0.1	0.4
	Chlorpromazine	GC–NPD, UPLC–PDA-CAD	0.05–0.1	0.1 (–0.5)
	Chlorprothixene	GC–NPD, LC–QqQ	0.02–0.1	0.3
	Clozapine	GC–NPD, UPLC–PDA-CAD	0.05–0.1	0.6
	Haloperidol	LC–QqQ	0.003	0.017
	Levomepromazine	GC–NPD, UPLC–PDA-CAD	0.1	0.025 (–0.2)
	Olanzapine	GC–NPD, UPLC–PDA-CAD	0.05–0.1	0.08
	Perphenazine	GC–MS, LC–QqQ	0.0002–0.005	0.0024
	Quetiapine	GC–NPD, UPLC–PDA-CAD	0.05–0.2	0.5
	Risperidone	LC–QqQ	0.0005–0.002	0.06
	Sulpiride	LC–QqQ, UPLC–PDA-CAD	0.02–0.05	0.4 (–1)
	Zuclopenthixol	GC–MS, LC–QqQ	0.005–0.01	0.05 (–0.1)

a GC, gas chromatography; NPD, nitrogen phosphorus detection; UPLC, ultra-performance liquid chromatography; PDA, photodiode array detection; CAD, charged aerosol detection; LC, liquid chromatography; QqQ, triple quadrupole mass spectrometry; MS, electron ionization mass spectrometry.

b LLOQ, lower limit of quantification: range applied over the study period.

c Upper end of trough plasma concentration range at steady state in living patients (ref. 9).

Two previously published approaches provide such reference concentration data that are well defined in terms of both investigation procedures and statistical treatment of results. In the grouped causes of death approach, also known as “the Druid approach” (12), the carefully evaluated cases were subdivided into poisonings by one specific substance only, multi-substance poisonings and non-poisoning controls, while concentration distributions in PM femoral blood were provided for each group. In the all causes of death approach (13, 14), summary statistics for drug concentrations in PM femoral blood were calculated without preselection of PM cases according to the cause of death or any criterion other than the sufficiently high number of qualified findings per each drug studied.

In this study, our objective was to add to the existing supply of reference concentration data by providing PM concentration distributions from large number of cases of fatal poisonings due to an antidepressant or an antipsychotic drug assigned as the principal intoxicant. Based on cases included in the Finnish national PM toxicology database between 2000 and 2020, summary statistics for drug concentrations in PM femoral blood (min, max, mean, 10th, 25th, 50th, 75th, 90th percentile) are reported for 17 antidepressant and for 12 antipsychotic drugs. In addition to this, the proportion of suicide, accident and undetermined manner of death is given for each drug. Consequently, the fatal concentration results are discussed by comparing with those previously published by the two different approaches, namely the grouped causes of death approach (15–17) and the all causes of death approach (18).

**Table II.** Descriptive Statistics for Fatal Antidepressant and Antipsychotic Drug Concentrations in Postmortem Femoral Blood according to Principal Drug Finding

	*N*	Min	Max	Mean	10th %ile	25th %ile	50th %ile	75th %ile	90th %ile
Antidepressants									
Amitriptyline	611	0.10	170	4.2	0.66	1.1	2.0	4.0	8.3
Bupropion	55	0.10	92	12	0.47	2.2	4.3	12	41
Citalopram	186	0.30	120	84	0.9	1.7	4.5	12	20
Clomipramine	42	0.30	8.4	2.1	0.51	0.9	1.6	2.7	3.8
Doxepin	346	0.05	40	4.9	0.65	1.2	2.6	6.3	12
Duloxetine	38	0.15	9	1.7	0.31	0.5	0.81	2.2	4.2
Fluoxetine	34	0.70	17	4.0	0.99	1.5	2.4	5.2	8.1
Fluvoxamine	10	6.4	30	14	6.8	7.3	9.1	20	23
Mianserin	31	0.10	13	1.9	0.40	0.50	0.90	1.9	4.0
Mirtazapine	178	0.30	48	3.9	0.60	0.93	1.9	4.0	8.0
Moclobemide	18	5.3	300	77	16	32	45	120	156
Nortriptyline	27	0.60	13	4.8	1.5	2.4	3.8	5.9	10
Paroxetine	76	0.04	55	2.5	0.24	0.45	0.90	1.7	4.7
Sertraline	33	0.40	17	2.7	0.60	0.93	1.9	2.9	5.6
Trazodone	8	0.20	27	13	1.1	3.5	10	25	26
Trimipramine	57	0.30	15	2.9	0.90	1.4	1.9	3.2	5.6
Venlafaxine	257	0.30	360	21	1.2	2.4	7.1	26	65
Antipsychotics									
Amisulpiride	2	2.9	33						
Chlorpromazine	29	0.20	29	4.6	0.66	1.1	2.1	6.5	8.5
Chlorprothixene	117	0.50	180	8.7	0.98	1.4	2.9	5.2	11
Clozapine	120	0.34	120	11	1.9	2.7	5.2	11	20
Haloperidol	6	0.17	1	0.48	0.23	0.31	0.43	0.56	0.80
Levomepromazine	478	0.10	220	3.4	0.40	0.79	1.5	2.9	5.3
Olanzapine	148	0.08	27	2.8	0.46	0.80	1.7	3.3	6.2
Perphenazine	6	0.08	540	90	0.085	0.13	0.26	0.60	270
Quetiapine	223	0.19	370	20	2.4	3.8	8.2	18	42
Risperidone	7	0.03	0.50	0.24	0.072	0.13	0.20	0.36	0.46
Sulpiride	19	1.5	70	15	3.2	4.7	7.8	14	41
Zuclopenthixol	6	0.36	1.2	0.72	0.43	0.51	0.57	0.98	1.2

Abbreviations: %ile, percentile; 50th %ile, median.

## Materials and methods

In Finland, all sudden and unexpected deaths undergo a medico-legal investigation process as required by the law. Most medico-legal cases include comprehensive PM toxicology as autopsy is performed in 16% and PM toxicology in 12% of all deaths in Finland. The PM interval from death to autopsy is on average 6 days. The toxicological examination of samples taken at autopsy include screening and quantification of hundreds of drugs and poisons by quality-assured methods in an accredited laboratory.

As part of a comprehensive PM toxicology panel (18), antidepressants and antipsychotics were screened in urine by ultra-high performance liquid chromatography coupled with high-resolution time-of-flight mass spectrometry (19). Quantification of antidepressants and antipsychotics in femoral blood was performed by dual‐column gas chromatography with nitrogen phosphorus detection (GC–NPD) (20) until 2014, and after that by ultra-performance liquid chromatography coupled with photodiode array detection and charged aerosol detection (UPLC–PDA-CAD) (21). For low-dose compounds, quantification was performed by gas chromatography–mass spectrometry (GC–MS), using selected ion monitoring, or by liquid chromatography–triple quadrupole mass spectrometry (LC–QqQ), using selected reaction monitoring. Concentration results that were above the measurement range were obtained by re-analysis of the respective samples after dilution. Median concentrations and percentiles were used to characterize the data.

Table I shows the analysis methods used for the antidepressant and antipsychotic drugs with the LLOQ range applied during the study period. For comparison, therapeutic reference concentrations in living subjects’ plasma according to Schulz et al. (9) are given.

Based on PM toxicology analyses, background information, autopsy findings and other possible evidence and consultation, the forensic pathologist determined the cause and manner of death in the death certificate. In fatal poisonings, the forensic pathologist additionally recorded on the death certificate all the substances that were involved in the poisoning and defined one principal drug finding of poisoning. PM toxicology results and information from the death certificate were then entered into the national PM toxicology database maintained by the Finnish Institute for Health and Welfare.

All fatal poisonings between 2000 and 2020 were retrieved from the Finnish national PM toxicology database in which the principal finding of poisoning, according to the forensic pathologist, was an antidepressant or an antipsychotic drug. These cases included both poly-drug and single drug poisonings. Cases where an antidepressant or an antipsychotic was involved in the cause of death but not as the principal finding were excluded.

The study was carried out based on the research permit THL/1922/6.02.00/2017, issued by the Finnish Institute for Health and Welfare, Finland.

## Results

Table II shows descriptive statistics for the concentrations of 17 antidepressants (*N* = 2,007) and 12 antipsychotics (*N* = 1,161) in fatal poisonings according to the principal drug finding, as determined in PM femoral blood. The most prevalent antidepressants in descending order were amitriptyline, doxepin, venlafaxine, citalopram and mirtazapine, and the most prevalent antipsychotics were levomepromazine, quetiapine, olanzapine, clozapine and chlorprothixene.

**Figure 1.**
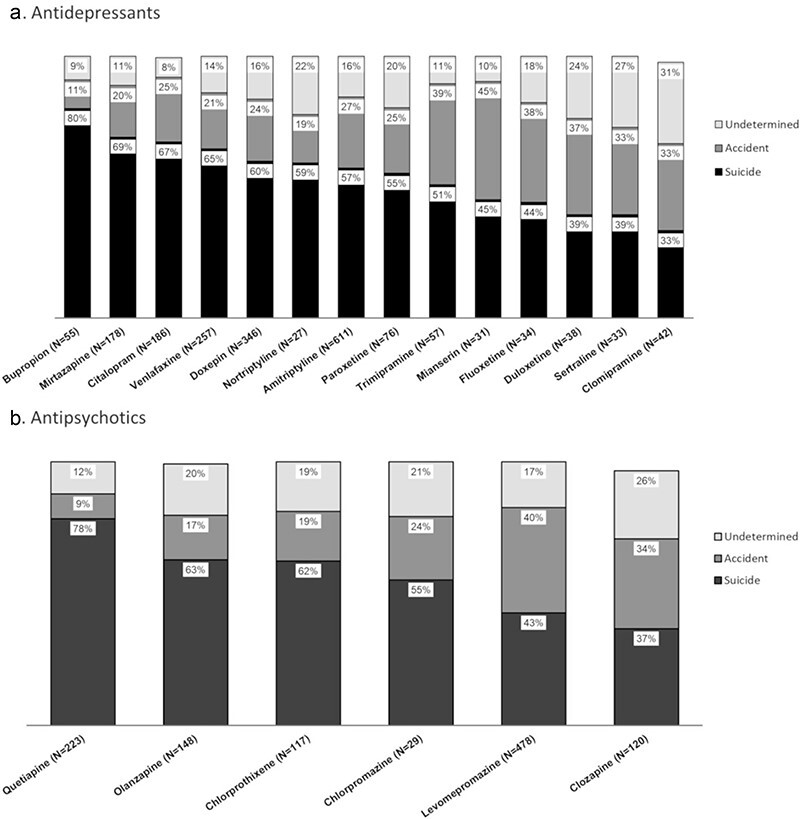
Proportions of suicide, accident and undetermined manner of death in fatal antidepressant and antipsychotic drug poisonings. Drugs with *N* <20 were excluded from graph.

The relative proportions of suicide, accident and undetermined manner of death in fatal antidepressant and antipsychotic drug poisonings for drugs with *N* ≥20 are shown in Figure 1.

## Discussion

We have provided descriptive statistics for fatal concentrations of commonly used antidepressant and antipsychotic drugs in PM femoral blood analyzed in one accredited laboratory. The data were based on large number of medico-legal cases in which the cause of death, according to the forensic pathologist, was poisoning due to an antidepressant or antipsychotic drug either alone or with concomitant substances. To evaluate the usability and general validity of the results obtained, we compared them with previously published results based on the established approaches discussed below.

**Figure 2.**
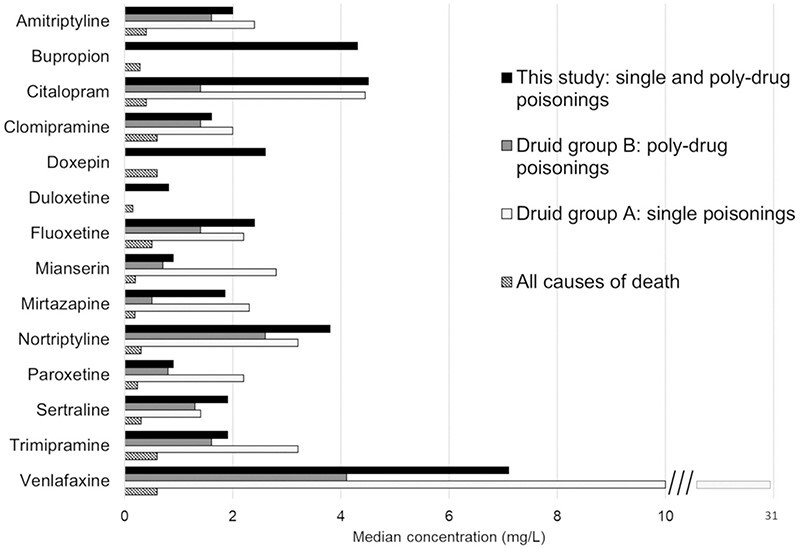
Comparison of median antidepressant drug concentrations in post-mortem femoral blood between this study and reference studies. Drugs with *N* <20 in this study were excluded from graph.

**Figure 3.**
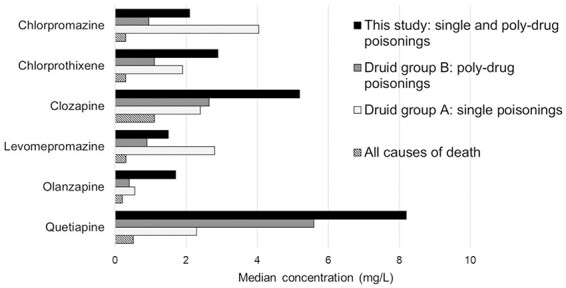
Comparison of median antipsychotic drug concentrations in post-mortem femoral blood between this study and reference studies. Drugs with *N* <20 in this study were excluded from graph.

Comparison of median fatal concentrations in PM femoral blood between this study and the Swedish reference studies is shown in Figures 2 and 3 for antidepressants and antipsychotics, respectively. The reference data consisted of published concentrations obtained by the Druid group B (poly-drug poisonings) and Druid group A (single drug poisonings) approach (15–17), the single-drug poisonings being regularly associated with higher concentrations. As an approximation of “normal” PM concentrations, Figures 2 and 3 also show published median concentrations relying on the all causes of death approach from a Finnish study (18). These “normal” PM concentrations were generally similar to the living patients’ plasma concentration ranges presented by Schulz (9), except for a couple of drugs: higher than twofold the plasma concentration was found for the antidepressants doxepin, fluvoxamine and paroxetine and for the antipsychotics chlorpromazine, levomepromazine and olanzapine, and lower than half the plasma concentration was seen for the antidepressant trazodone and the antipsychotic haloperidol.

The median fatal drug concentrations in this study often fall between the Druid group B (poly-drug poisonings) and Druid group A (single drug poisonings) reference concentrations, as expected. However, this regularity does not apply to all drugs, and although there may be several explanations for this behavior, one reason is the small number (*N* <10) of Druid group A cases for some antidepressants (fluoxetine, mianserin, mirtazapine, paroxetine, sertraline) and antipsychotics (chlorpromazine, chlorprothixene, quetiapine). The fatal concentrations can in any case be clearly distinguished from the “normal” concentrations: median fatal antidepressant and antipsychotic concentrations in this study were on average (median) 9.3 (6.3) and 18 (9.8) times higher than the “normal” PM concentrations, respectively.

**Figure 4.**
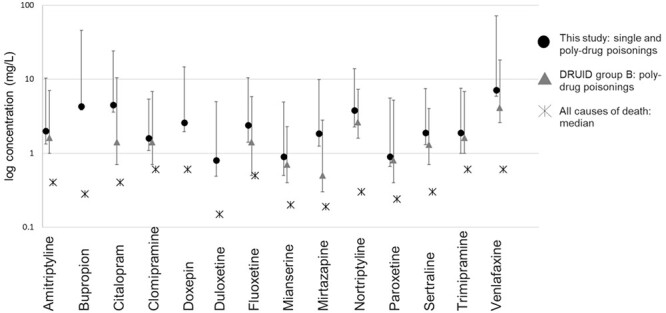
Comparison of median antidepressant drug concentrations in post-mortem femoral blood between this study and reference studies on logarithmic scale. Whiskers represent 10th and 90th percentiles, where available. Drugs with *N* <20 in this study were excluded from graph.

**Figure 5.**
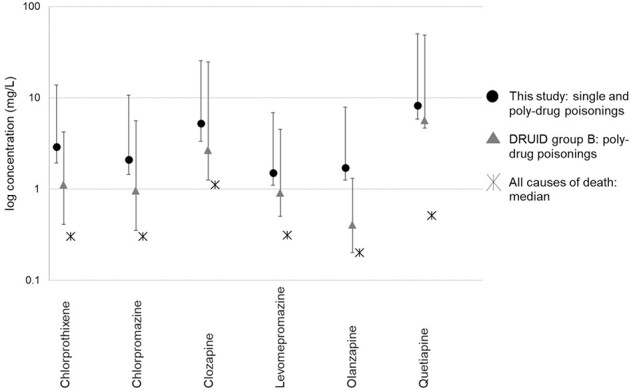
Comparison of median antipsychotic drug concentrations in post-mortem femoral blood between this study and reference studies on logarithmic scale. Whiskers represent 10th and 90th percentiles, where available. Drugs with *N* <20 in this study were excluded from graph.

Comparison of the ranges of fatal concentrations in PM femoral blood (10th percentile, median, 90th percentile on logarithmic scale) between this study and the Druid group B approach is illustrated in Figures 4 and 5 for antidepressants and antipsychotics, respectively, while “normal” PM concentrations were based on the all causes of death approach as described above. Today, most poisonings are poly-drug poisonings, and consequently comparison of these groups is appropriate.

Summary statistics relying on extensive data generally produce better evaluable data for fatal concentrations than individual case reports. The concentrations referenced in the Baselt’s handbook (11), or any other compilation, are usually based on much fewer number of cases than in this study, and consequently are less representative. Moreover, medians are better indicators than means for describing skewed distributions. There may also be publication bias associated with case reports, which means that exceptionally high concentrations are overrepresented in the published papers. Baselt’s handbook (11) gives mean fatal concentration of 3.8 mg/L for the antidepressant fluoxetine and 18 mg/L for the antipsychotic quetiapine, the values being comparable to the means 4.0 and 20 mg/L but higher than the medians 2.4 and 8.2 mg/L in this study, respectively.

It is difficult to assess the extent to which PM redistribution and drug stability issues are involved in the concentrations presented in this study. Mantinieks et al. (22) used paired AM specimen and PM mortuary admission femoral blood drug concentrations to construct a retrospective compilation of PM/AM drug concentration ratios. The median PM/AM ratios for all antidepressants were >1 but the antipsychotics showed varying behavior. These authors concluded that while the median PM/AM ratios demonstrated some drug-dependent trends, there was no obvious relationship between AM specimens and PM femoral blood taken at mortuary admission due to case-to-case variation.

The relative proportion of suicides depicted in Figure 1 shows marked differences between individual drugs, being higher than 50% of cases for 9 out of 14 antidepressants and 4 out of 7 antipsychotics. Bupropion stands out with the highest suicide rate among antidepressants in this study, while related anecdotal information has been presented in previous studies (23–25). For the rest of the antidepressants, the overall picture is less clear. As for the antipsychotics, the pronounced role of quetiapine and olanzapine in suicides has been noticed also elsewhere (26). Clozapine, on the other hand, is associated with a low proportion of suicides in this study. Clozapine therapy has demonstrated superiority in preventing suicide attempts in patients with schizophrenia and schizoaffective disorder who generally are at high risk for suicide (27). The relatively large share of undetermined manner of death in this study demonstrates the difficulty for the forensic pathologist to make a choice between suicide and accidental death both in antidepressant and antipsychotic poisonings in the absence of a credible suicide note. However, deaths of undetermined intent have previously been included in the category of suicides (26).

In terms of fatal toxicity, i.e., the ratio of the number of drug-related deaths to the consumption or overdoses of the drug, the older TCAs are more toxic in overdose than the SSRIs, while most other types of antidepressants fall in between these groups (3, 28–30). Antipsychotic deaths are accumulated on fewer substances compared to antidepressants. Fatal toxicity of the first-generation antipsychotics seems to be higher than that of the second-generation drugs (3). Clozapine, although being a second-generation antipsychotic, possesses high toxicity and therefore pharmacotherapy should be carefully controlled. Clozapine is usually used to treat patients with schizophrenia who have not responded to other antipsychotics and patients who may already be at higher risk of suicide (26).

This study has limitations. First, during the study period, the LLOQ was not always sufficiently low to cover low therapeutic concentrations for some drugs, particularly bupropion, mianserin, paroxetine and olanzapine. However, the clinical reference concentrations cited in Table I represent the upper end of trough plasma concentration range at steady state in living patients (9), whereas therapeutic concentrations for these drugs in PM blood can be higher due to PM redistribution. While the LLOQ is an important factor relative to the statistical concentration distribution (18), it is estimated to have only a minor effect on the “normal” concentrations presented and a negligible effect on the median fatal concentrations.

Second, due to the summary nature of the research, it was not possible to address the factors that affect the concentrations of individual drugs, such as the PM redistribution phenomenon, drug stability, drug interactions and pharmacogenetics, let alone the case characteristics. Particularly, escitalopram was not analyzed enantioselectively, and consequently the citalopram concentrations reflect the use of both racemic citalopram and escitalopram. However, only 27% lower plasma concentrations were listed for escitalopram than citalopram in the therapeutic drug monitoring guidelines (31), suggesting that the lack of chiral analysis has little effect on the results of this study. Another issue is associated with bupropion because of the instability of the parent compound (25). Drug metabolites were not included in this study due to insufficient data available, but will be considered in future studies.

Finally, there is also the risk that the diagnosis given by the forensic pathologist is incorrect in some cases and especially defining the principal finding of fatal poisoning can be difficult. There is also the danger of circular reasoning because lethal reference concentrations have been available in the literature for a long time. However, the length of the study period and the large number of cases as well as the considerable number of forensic pathologists that were in charge obviously compensate for individual incorrect diagnoses.

## Conclusions

The extensive PM reference concentration data presented in this study, combined with similar information from previous studies, help the forensic toxicologist and the forensic pathologist evaluate the possibility of antidepressant or antipsychotic poisoning as the cause of death. Despite the wide range of fatal concentrations associated with each drug, the concentration percentiles reported here can be used to rationalize case interpretation with the goal of evidence-based forensic toxicology. However, the summary statistics should only be considered as an aid in the interpretation of individual cases, where all other information related to the case must be taken into account.

## Data Availability

The data underlying this article cannot be shared publicly due to the terms of the research agreement.
